# Participation of under-represented communities in an online cognitive ageing platform and predictors of willingness to be contacted for future research

**DOI:** 10.1136/bmjph-2024-001721

**Published:** 2025-06-20

**Authors:** Katherine D Ellingson, William J Degnan, Tomas Nuño, Ashleigh Horton, Carolina Carrasco, Melanie Rubio, Yunjia Yang, Matthew D DeBoth, Megan Johnson, Harshini Venkatachalam, Bonnie J LaFleur, Lee Ryan, Carol A Barnes, David W Coon, Matthew Huentelman, Zhao Chen, Precision Aging Network (PAN), A Aldabergenova

**Affiliations:** 1 The University of Arizona Mel and Enid Zuckerman College of Public Health, Tucson, Arizona, USA; 2 The Translational Genomics Research Institute (TGen), Phoenix, Arizona, USA; 3 The University of Arizona College of Pharmacy, Tucson, Arizona, USA; 4 The University of Arizona College of Science, Tucson, Arizona, USA; 5 Arizona State University Edson College of Nursing and Health Innovation, Phoenix, Arizona, USA

**Keywords:** methods, Community Health, Age Factors

## Abstract

**ABSTRACT:**

**Background:**

Black and Hispanic communities are under-represented in cognitive ageing research. MindCrowd is an online platform that offers interactive cognitive games and facilitates research. We sought to assess participation in MindCrowd and willingness to be contacted for future studies by race, ethnicity and other demographic variables.

**Methods:**

Adults were recruited into MindCrowd through national media campaigns and enhanced local engagement (ELE) in and around four cities with robust black (Atlanta and Baltimore) and Hispanic (Miami and Tucson) populations. Recruiting in ELE regions involved direct contact with potential participants via community forums or established research cohorts. Participation was defined as completing two 5-minute cognitive games and a demographic questionnaire, and participation incidence was calculated per 100 000 adults using census data and was compared by region using incidence rate ratios (IRRs). Willingness to be contacted for future research was defined as participants submitting an email address for researcher follow-up and was modelled as a binary outcome using logistic regression to generate adjusted ORs (aORs) for age, sex, region, race and ethnicity.

**Results:**

From 17 June 2022 to 28 December 2023, 49 934 adults participated in MindCrowd. Nationwide, the majority were female (87.2%) and >50 years old (75.2%). In ELE regions, 8.4% of participants were black versus 2.3% in non-ELE regions; 21.8% were Hispanic in ELE regions versus 16.4% in non-ELE regions. MindCrowd participation incidence was higher in ELE than non-ELE regions (32.4 vs 17.9 per 100 000 adults; IRR=1.78, 95% CI, 1.73 to 1.83). Overall, 42.1% of participants nationwide were willing to be contacted for future research, with elevated odds for all ELE regions (vs collective non-ELE regions). Compared with white participants, black participants had 23% higher odds of willingness to be contacted (aOR=1.23; 95% CI, 1.10 to 1.36); compared with non-Hispanic participants, Hispanic participants had 20% higher odds (aOR=1.20; 95% CI, 1.13 to 1.27).

**Conclusions:**

Following nationwide and regional efforts to recruit a diverse participant pool into the online platform MindCrowd, regions with ELE had higher rates of participation than the rest of the nation across race and ethnicity categories. Controlling for region, black and Hispanic individuals showed marginal but statistically elevated willingness to be contacted for future research.

**WHAT IS ALREADY KNOWN ON THIS TOPIC**

- Black and Hispanic communities will experience disproportionate increases in age-related cognitive decline in the coming decades but are under-represented in cognitive ageing research. This study examined participation in MindCrowd, an interactive online cognitive ageing platform, and subsequent willingness to be contacted for future research by race and ethnicity.

**WHAT THIS STUDY ADDS**

- Participation in MindCrowd was higher in regions where investments were made in local engagement (Atlanta, Baltimore, Miami and Tucson), although over 75% of participants in these regions were white and non-Hispanic. Once engaged in MindCrowd, black and Hispanic participants were marginally more likely to leave contact information for future research than white and non-Hispanic participants.

**HOW THIS STUDY MIGHT AFFECT RESEARCH, PRACTICE OR POLICY**

- Findings underscore ongoing challenges in representation of racially and ethnically diverse populations in ageing research but suggest that targeted campaigns can improve willingness to engage these populations in future research.

## Introduction

 Black and Hispanic individuals are under-represented in cognitive ageing research despite their elevated risk of early morbidity, mortality and age-related cognitive decline.^1^,^5^ Between 2020 and 2060, as the US population ages and diversifies, the estimated number of people living with Alzheimer’s disease will increase from 6.1 million to 13.9 million, reflecting increases of 63% among white, 192% among black and 423% among Hispanic individuals.^6^,^9^ These projections pose threats to the quality of life and economic stability for older adults and their caretakers, with the costs of formal and informal care estimated to reach US$1.4 trillion by 2060.^10^ Addressing the overall burden and inequities associated with ageing requires an evidence base to support interventions that promote cognitive health and prevent disease in a diverse population. There is a shortfall of black and Hispanic research participants in studies of cognitive health and ageing in general.^10 11^

Researchers have long documented challenges in recruiting individuals from minoritised populations into observational and interventional studies of older adults, but some have noted promising strategies, including engagement of community and caregiver networks and utilisation of culturally tailored messaging.^12^,^16^ Limited internet access and usage have been cited as barriers to engage older populations via online recruitment,^17^ but more recent studies have cited social media platforms as valuable in efficiently tailoring messages to reach hard-to-reach communities, including older adults.^18^,^21^ A 2024 review of online participation in cognitive testing among a large Norwegian cohort found that women aged 50–79 years were best represented, which aligns with other studies that suggest older age is less of a barrier to online research than social factors.^22^ The COVID-19 pandemic diminished opportunities for inperson recruitment, which incentivised researchers to use a range of creative strategies for the engagement of diverse populations in online research.^23 24^ Collectively, the scientific literature suggests that recruiting older adults into online research may benefit from inperson assistance and demonstrations with online platforms, as well as promotion of participation in online research through community liaisons, trusted clinical networks and established support networks.^18 25 26^

MindCrowd is an online platform designed to engage adults in games and educational content related to ageing and cognitive health. The platform launched in 2013 and has evolved over time to host multiple substudies. In 2021, the University of Arizona received funding from the National Institutes of Health to develop the Precision Aging Network (PAN), which supported a multisite initiative to host multiple cognitive ageing studies involving both cross-sectional and longitudinal research with the goal of overenrolling black and Hispanic individuals. In June 2022, MindCrowd launched a new website with an updated interface and enhanced functionality to allow people to join the study using different types of electronic devices including computers, tablets and smart phones. This study examines engagement patterns subsequent to the launch of the updated MindCrowd website in 2022.

The PAN researchers designed and implemented traditional media (radio, television and print) and social media campaigns tailored to black and Hispanic communities nationwide. Additionally, the PAN invested in regional teams to implement inperson activities in strategically selected regions with demographically diverse communities and established academic ageing research programmes. Research teams in Atlanta, Georgia (Emory University); Baltimore, Maryland (Johns Hopkins University); Miami, Florida (University of Miami); and Tucson, Arizona (University of Arizona) implemented a range of local recruiting activities and events to promote participation in MindCrowd. The objectives of this study were to: (1) determine if enhanced local engagement (ELE) in specific geographic regions could augment MindCrowd participation among black and Hispanic adults; (2) identify predictors of MindCrowd participant willingness to leave contact information for future research including age, region, sex, race and ethnicity.

## Methods

### Overview and public engagement

Researchers from the PAN sought to engage adults in MindCrowd, a web-based study on cognitive health and documented activities per established criteria for improving reporting of patient and public involvement in research.^27^ Members of the public and other stakeholders were involved in the initial concept and design of the MindCrowd platform by providing feedback to designers through targeted focus groups to inform user experience and research priorities. Ongoing feedback has been incorporated since the initial platform launch in 2013 and relaunch in 2022, through social media platforms and periodic focus groups. Specifically, comments from members of the public have led to alterations in the platform to streamline the user experience to decrease burden. Further, members of the public are encouraged to share their stories and experiences through MindCrowd’s social media channels and blog, which were considered by researchers in developing research questions. The public will be able to access study findings from this study through MindCrowd’s social media channels, blogs and newsletters.

The PAN had a goal of maximising recruitment of all adults, with enhanced recruiting of adults >50 years and those identifying as black or Hispanic. Nationwide, researchers promoted participation in MindCrowd via traditional campaigns in radio, television and print media involving press interviews and advertising. The PAN also employed social media campaigns informed by marketing professionals and targeted towards black, Hispanic, older and male social media users. Men were targeted based on proportionally low male engagement in Mindcrowd, which is well established in the recruitment and retention literature.^28^,^30^ ELE efforts were implemented in four regions intentionally selected for race or ethnic diversity and location near a major academic centre. The populations encompassed in the four ELE regions were defined as adults with residential zip codes located within 60 miles of Emory University (Atlanta), Johns Hopkins University (Baltimore), University of Miami (Miami) and University of Arizona (Tucson). These ELE regions collectively encompassed 7% (n=18 407 102) of the US adult population. Examples of local recruiting activities included tabling at or hosting community events, visits to community and senior centres, free lectures and distribution of educational materials, holding virtual events and engagement of individuals already affiliated with university research cohorts. All national and regional recruiting activities received Institutional Review Board approval from Western Institutional Review Board (WIRB)-Copernicus Group (WCG) (protocol #20215906). The approval covers the consent language presented to prospective MindCrowd participants, after they visit the website and before they begin two 5-minute MindCrowd cognitive games online.

Each of the four ELE regions took slightly different approaches to introducing individuals to MindCrowd. Teams in all ELE regions emailed or called individuals who had agreed to be contacted by university research teams for cognitive health research, either as part of a current or previous study. Each region also hosted or tabled at community events to promote PAN and introduce MindCrowd. Atlanta and Baltimore had more robust ageing cohorts to draw prospective participants, whereas Miami and Tucson relied more on inperson and virtual events to promote MindCrowd. The Miami ELE conducted additional virtual events to engage a broader population, and the Tucson ELE employed undergraduate and graduate student ambassadors from the University of Arizona to support engagement through community events and visits to senior and community centres. Student ambassadors also helped older adults with the technology interface by helping them log onto the platform via laptop or computer. Recruiting teams from the four ELE regions met weekly via Zoom to discuss challenges and share strategies and successes.

### Study design and setting

This study design was cross-sectional and considered MindCrowd engagement data from 17 June 2022 to 28 December 2023, which corresponds with the launch of the enhanced MindCrowd platform to the date of analysis. Although available worldwide during this time period, this study focused on US participation in MindCrowd and therefore excluded any non-US or missing zip codes. ‘Participation’ was defined as completion of two 5-minute assessments, including a memory test (paired associates learning test)^31^ and a simple visual reaction time test,^32^ as well as a short demographic questionnaire. After consenting and before accessing the two online assessments, individuals were advised to complete the assessments in a place with minimal distractions. For participants recruited via inperson events in ELE regions, a quiet space was provided on-site, if possible, for real-time participation. Teams in ELE regions modified recruiting practices based on direct participant feedback throughout inperson or phone follow-up recruiting processes. Since August 2023, those engaging with MindCrowd had the option to take the assessment and demographic questionnaire in Spanish.

Once finished with the two online MindCrowd assessments, individuals were directed to a demographic questionnaire, which included fields for sex, race, ethnicity, age and residential zip code. Participants had to submit the questionnaire to see the results from their two cognitive tasks, which included a peer comparison score. Participants were required to complete the age and sex fields to complete the questionnaire, but they had the option to leave race, ethnicity and zip code blank. PAN researchers made the decision to require age and sex fields based on the need to adjust scores on the two cognitive assessments by age and sex. For this study, researchers excluded those with invalid or non-US zip codes because defining regions as ELE versus non-ELE was critical to the study question.

For the sex field on the demographic questionnaire, participants were asked to report sex as male, female or intersex. For race, participants were asked to self-identify as American Indian/Alaska Native, Asian, black/African American, Native Hawaiian/other Pacific Islander, multiracial/mixed Race, white or ‘Prefer not to answer’. For ethnicity, participants were asked to self-identify as Hispanic/Latino, non-Hispanic/Latino or ’Prefer not to answer’. Race and ethnicity were asked separately.

At the end of the demographic questionnaire, participants were given the option to leave their email address to be contacted for future research. If an email address was submitted, the individual was designated as ‘willing to be contacted for future research’. This study considered willingness to be contacted as a binary outcome.

### Data source and statistical analysis

Zip code tabulation areas from the US census data were used to define the population estimates around point-based zip codes using 2020 tabulation blocks. These parameters were used to define population denominators for the ELE regions, non-ELE regions and the nation. Since MindCrowd targets adults only and regions with larger young populations could bias participation incidence downwards, the proportions of individuals 18 years of age or older from the July 2022 census in each region were applied to population denominators to generate MindCrowd participation per 100 000 adults nationwide, for ELE regions and for non-ELE regions.^33 34^ Incidence rate ratios (IRRs) were calculated for participation in each ELE relative to all non-ELE regions combined. Two methods were used to calculate CIs, including the large sample normal approximation and Poisson regression models.^35 36^

MindCrowd participants who had completed the two cognitive tasks and demographic questionnaire were asked if they were willing to leave their email for more information and involvement in future follow-up studies. Willingness to be contacted for future research was characterised by whether the participant left their email address or not. χ^2^ tests for independence were used to assess unadjusted associations between demographic variables and willingness to be contacted. Logistic regression was used to model willingness to be contacted in a fully-adjusted multivariable context. Adjusted ORs (aORs) were generated for regions (ELE region vs non-ELE region), 10-year age categories (with <50 as reference), sex (female as reference), race (white as reference) and ethnicity (non-Hispanic as reference). Researchers included all variables in adjusted analyses to determine the independent effect of each variable owing to potential confounding of race and ethnicity by region, sex and age. All statistical analyses were conducted in SAS V.9.4.

## Results

From 17 June 2022 to 28 December 2023, 49 934 adults participated in MindCrowd. The majority were female (87.2%), white (83.7%), non-Hispanic (80.5%) and >50 years old (75.2%). Nationwide, 3.1% of participants identified as black, with 8.4% in ELE regions (13.0% in Atlanta and 11.7% in Baltimore) and 2.3% in non-ELE regions (table 1). Overall, 17.1% of participants identified as Hispanic nationwide with 21.8% in ELE regions (56.5% in Miami and 22.1% in Tucson) and 16.4% in non-ELE regions (table 1). Of those who identified as American Indian/Alaska Native, 31.8% identified as Hispanic, and of those who identified as Native Hawaiian/Other Pacific Islander, 32.2% identified as Hispanic. Of note, of those who preferred not to answer the race question, 54.4% identified as Hispanic.

**Table 1:** Demographics of MindCrowd participants nationwide (n=49 934) who accessed the online platform and completed two 5-minute memory and attention tasks in four enhanced local engagement regions within 60 miles of academic centres in Atlanta, Baltimore, Miami and Tucson (n=6295) and in all other regions of the USA (n=43 639) from 17 June 2022 through 28 December 2023*

	Nationwide	Enhanced local engagement regions combined	Enhanced engagement regions stratified				All other (non-ELE) regions of the USA combined†
	n=49 934	n=6295	Atlantan=1425	Baltimoren=1981	Miamin=1483	Tucsonn=1406	n=43 639
Age	n (col %)	n (col %)	n (col %)	n (col %)	n (col %)	n (col %)	n (col %)
<50	12 383 (24.8)	1611 (25.6)	260 (18.3)	433 (21.9)	413 (27.9)	505 (35.9)	10 772 (24.7)
50–59	13 294 (26.6)	1576 (25.0)	400 (28.1)	577 (29.1)	409 (27.8)	190 (13.5)	11 718 (26.9)
60–69	15 232 (30.5)	1844 (29.3)	477 (33.5)	616 (31.1)	408 (27.5)	343 (24.4)	13 388 (30.7)
70–79	7721 (15.4)	1075 (17.1)	252 (17.7)	299 (15.1)	219 (14.8)	305 (21.7)	6646 (15.2)
80+	1304 (2.6)	189 (3.0)	36 (2.5)	56 (2.8)	34 (2.3)	63 (4.5)	1115 (2.6)
Sex							
Male	6350 (12.7)	1097 (17.4)	198 (13.9)	309 (15.6)	259 (17.5)	331 (23.5)	5253 (12.0)
Female	43 544 (87.2)	5193 (82.5)	1225 (86.0)	1671 (84.4)	1222 (82.4)	1075 (76.5)	38 351 (87.9)
Intersex	40 (0.1)	5 (0.1)	2 (0.1)	1 (0.1)	2 (0.1)	0 (0)	35 (0.1)
Race*							
Am Indian/Alaska Native	582 (1.2)	70 (1.2)	19 (1.4)	15 (0.8)	9 (0.7)	27 (2.0)	512 (1.3)
Asian	1099 (2.3)	158 (2.7)	22 (1.7)	54 (2.9)	16 (1.2)	66 (4.9)	941 (23)
Black	1452 (3.1)	500 (8.4)	174 (13.0)	219 (11.7)	73 (5.4)	34 (2.5)	950 (2.3)
Native Hawaiian/Pacific Islander	153 (0.3)	14 (0.2)	0 (0.0)	6 (0.3)	0 (0.0)	8 (0.6)	139 (0.3)
Multiracial/mixed race	1964 (4.2)	271 (4.6)	30 (2.3)	67 (3.6)	115 (8.4)	59 (4.4)	1693 (4.1)
Prefer not to answer†	2380 (5.1)	239 (4.0)	42 (3.1)	63 (3.4)	71 (5.2)	63 (4.7)	2141 (5.2)
White	39 288 (83.7)	4672 (78.9)	1049 (78.5)	1445 (77.3)	1081 (79.2)	1097 (81.0)	34 616 (84.4)
Ethnicity*							
Hispanic	8238 (17.1)	1330 (21.8)	80 (5.8)	140 (7.3)	809 (56.5)	301 (22.1)	6908 (16.4)
Prefer not to answer†	1187 (2.5)	140 (2.3)	30 (2.2)	34 (1.8)	43 (3.0)	33 (2.4)	1047 (2.5)
Non-Hispanic	38 830 (80.5)	4634 (75.9)	1276 (92.1)	1743 (90.9)	579 (40.5)	1026 (75.4)	34 206 (81.1)
Contactable							
Yes	21 005 (42.1)	3086 (49.0)	674 (47.3)	891 (45.0)	723 (48.8)	798 (56.8)	17 919 (41.1)
No	28 929 (57.9)	3209 (51.0)	751 (52.7)	1090 (55.0)	760 (51.3)	608 (43.2)	25 720 (58.9)

* Considered a non-missing response.

† Nationwide there were 6.0% missing race data (n=3016) and 3.4% (n=1679) missing ethnicity data; there were no missing data for age or sex.

Nationwide participation in MindCrowd was 19.1 per 100 000 adults in the USA (table 2). Within the four ELE regions, MindCrowd participation incidence was significantly higher (34.2 per 100 000 adults) than in the rest of the rest of the nation (17.9 per 100 000 adults). In the four ELE regions, IRRs comparing participation with non-ELE regions ranged from 1.47 (95% CI, 1.40 to 1.55) for Baltimore to 8.40 (95% CI, 7.88 to 8.76) for Tucson (table 2). Participation incidence for each ELE region was statistically higher (p<0.001 for each regional comparison) than for the non-ELE regions combined.

**Table 2:** MindCrowd participation rates, defined as the number of individuals who accessed the online platform and completed two 5-minute memory and attention tasks divided by US adult population nationwide and in four enhanced local engagement regions within 60 miles of academic centres in Atlanta, Baltimore, Miami and Tucson^33 34^

	MindCrowd participants	Estimated adult population	Participation per 100K adults	Participation incidence rate ratio (IRR)	Lower CI for IRR	Upper CI for IRR
Nationwide	49 934	261 808 482	19.1	IRR	LCL IRR	UCL IRR
Enhanced local engagement regions (all)	6295	18 407 102	34.2	1.91	1.86	1.96
Atlanta	1425	5 394 668	26.4	1.47	1.40	1.55
Baltimore	1981	7 487 106	26.5	1.48	1.41	1.54
Miami	1483	4 581 613	32.4	1.81	1.71	1.90
Tucson	1406	943 714	149.0	8.31	7.88	8.76
Non-enhanced local engagement regions	43 639	243 401 380	17.9	Reference		

LCL, lower confidence level; UCL, upper confidence level.

Nationwide, 42.1% of participants left their email addresses to be contacted for future research; in the four ELE regions combined, 49.0% of participants left contact information versus 41.1% in all other regions of the USA (table 3). Among black participants, 48.4% left contact information nationwide, with higher proportions leaving contact information in all local engagement regions, including 61.5% in Atlanta, 59.8% in Baltimore, 52.1% in Miami and 64.7% in Tucson (table 3). Among Hispanic participants, 45.6% left contact information nationwide, with higher proportions leaving contact information in Miami (53.5%) and Tucson (51.8%).

**Table 3:** Counts of participants willing to be contacted for future research (ie, ‘contactable’) and per cent willing to be contacted (vs declining to be contacted) nationwide, in four enhanced engagement regions within 60 miles of academic centres in Atlanta, Baltimore, Miami and Tucson and in all other regions of the USA from 17 June 2022 through 28 December 2023

	Nationwide*	Enhanced local engagement (ELE) regions combined*	Enhanced engagement regions stratified*				All other (non-ELE) regions of the USA combined*
	n (%)	n (%)	Atlantan (%)	Baltimoren (%)	Miamin (%)	Tucsonn (%)	n (%)
Total contactable	21 005 (42.1%)	3086 (49.0%)	674 (47.3%)	891 (45.0%)	723 (48.8%)	798 (56.8%)	17 919 (41.1%)
Age	†	†	†		†	†	†
<50	4571 (36.9)	646 (40.1)	105 (40.4)	156 (36.0)	172 (41.7)	213 (42.2)	3925 (36.4)
50–59	5763 (43.4)	777 (49.3)	179 (44.8)	278 (48.23)	209 (51.1)	111 (58.4)	4986 (42.6)
60–69	6646 (43.6)	957 (51.9)	248 (52.0)	272 (44.2)	205 (50.3)	232 (67.6)	5689 (42.5)
70–79	3450 (44.7)	606 (56.4)	129 (51.2)	158 (52.8)	121 (55.3)	198 (64.9)	2844 (42.8)
80+	575 (44.1)	100 (52.9)	13 (36.1)	27 (48.2)	16 (47.1)	44 (69.8)	475 (42.6)
Sex	†						†
Male	2509 (39.5)	549 (50.1)	92 (46.5)	144 (46.6)	130 (50.2)	183 (55.3)	1960 (37.1)
Female	18 475 (42.4)	2534 (48.8)	581 (47.4)	746 (44.6)	592 (48.5)	615 (57.2)	15 941 (41.6)
Intersex	21 (52.5)	3 (60.0)	1 (50.0)	1 (100.0)	1 (50.0)		18 (51.4)
Race†	†	†	†	†	†	†	†
American Indian/Alaska Native	229 (39.4)	37 (52.9)	10 (52.6)	4 (26.7)	4 (44.4)	19 (70.4)	192 (37.5)
Asian	375 (34.1)	70 (44.3)	12 (54.6)	20 (37.0)	8 (50.0)	30 (45.5)	305 (32.4)
Black	702 (48.4)	298 (59.6)	107 (61.5)	131 (59.8)	38 (52.1)	22 (64.7)	404 (42.4)
Native Hawaiian/Pacific Islander	63 (41.2)	3 (21.4)	–	–	–	3 (37.5)	60 (43.2)
Multiracial/mixed race	951 (48.4)	140 (51.7)	16 (53.3)	33 (49.3)	59 (51.3)	32 (54.2)	811 (47.9)
Prefer not to answer*	764 (32.1)	85 (35.6)	13 (31.0)	17 (27.0)	28 (39.4)	27 (42.9)	679 (31.7)
White	17 195 (43.8)	2353 (50.4)	490 (46.7)	664 (46.0)	545 (50.4)	654 (59.6)	14 842 (42.9)
Ethnicity†		†			†	†	†
Hispanic	3753 (45.6)	683 (51.4)	36 (45.0)	61 (43.6)	443 (53.5)	153 (50.8)	3070 (44.4)
Prefer not to answer*	299 (25.2)	44 (31.4)	6 (20.0)	8 (23.5)	14 (32.6)	16 (48.5)	255 (24.4)
Non-Hispanic	16 701 (43.0)	2322 (50.2)	623 (48.8)	814 (46.7)	269 (46.5)	616 (60.0)	14 379 (42.0)

* Counts (n) represent the number contactable and the % contactable; the n for non-contactable is not shown.

† Indicates statistically significant p value at the alpha=0.05 level of significance. P values were computed from Pearson’s χ2 test for contactable versus non-contactable counts. For variables having cells with expected counts <5, p values were computed from Fisher’s exact test.

For all age groups, the proportion of participants willing to leave contact information was greater in the four ELE regions combined. Of note, among white participants, willingness to leave contact information was higher in the enhanced local recruitment regions (50.4%) compared with the rest of the nation (42.9%). Nationwide and in ELE regions, participants 50 years of age and older were more likely to leave contact information for follow-up studies compared with those under 50. Those most likely to leave contact information were in the 70–79 year-old category (44.7% nationwide, 56.0% for ELE regions and 42.8% for the rest of the USA).

**Figure 1:**
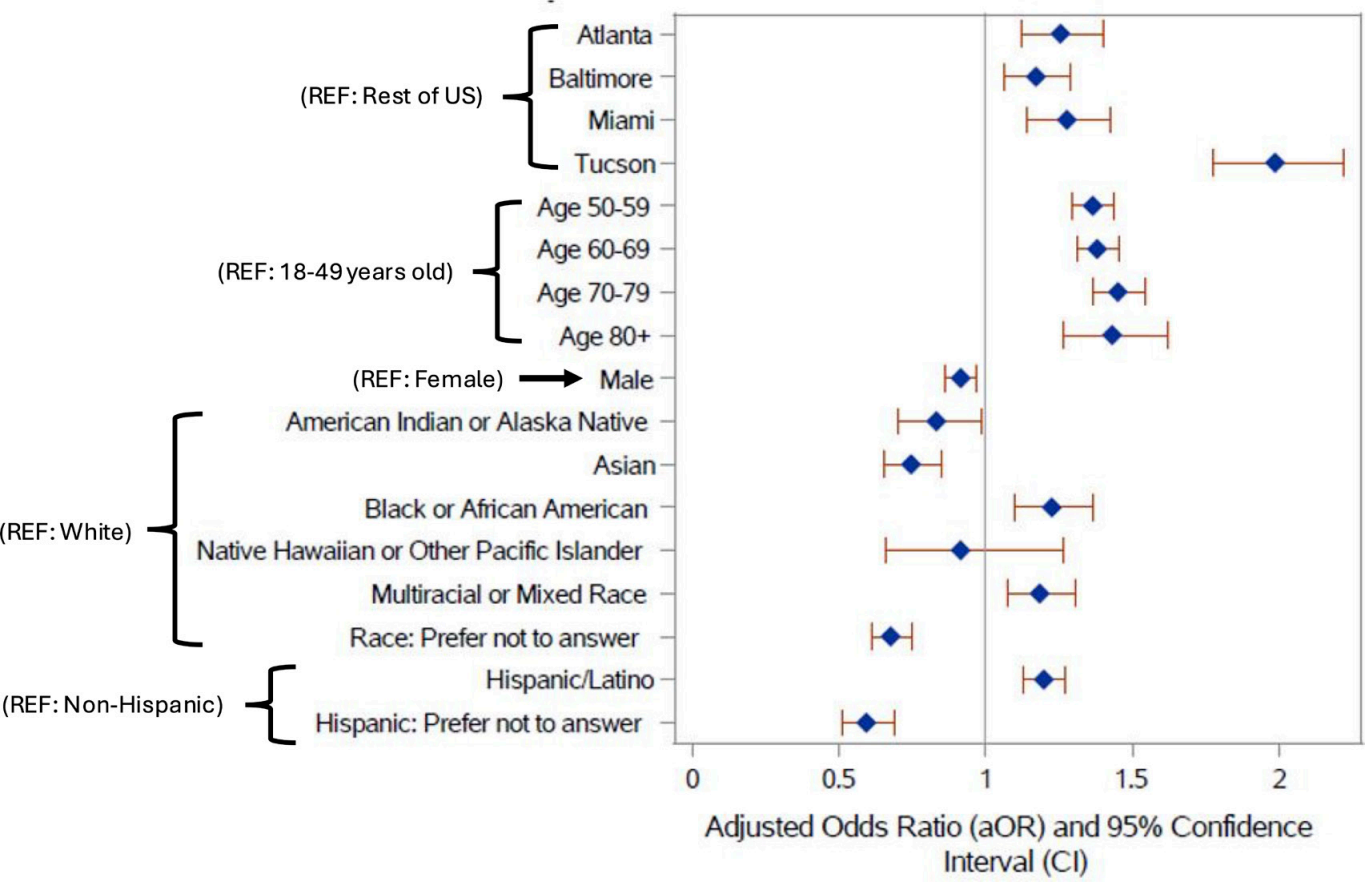
Forest plot of adjusted odds ratios (blue diamonds) with 95% CI (red lines) generated from a multivariable logistic regression for participant willingness to be contacted for future research by enhanced local engagement region (reference = rest of the USA), age (reference = 18–49 years old), sex (reference = female), race (reference = white) and ethnicity (reference = Non-Hispanic) from 17 June 2022 to 28 December 2023.

According to multivariable logistic regression modelling, willingness to be contacted for future research revealed participant characteristics associated with statistically higher odds of leaving contact information for future follow-up (figure 1). Participants in Atlanta, Baltimore, Miami and Tucson ELE regions collectively had 46% higher odds of leaving their contact information (aOR=1.46; 95% CI, 1.30 to 1.62). In the multivariable model examining each ELE region individually relative to all non-ELE regions in the USA, participants in Atlanta had 26% higher odds of leaving contact information (aOR=1.26; 95% CI, 1.12 to 1.40), Baltimore had 17% higher odds (aOR=1.17; 95% CI, 1.07 to 1.29), Miami had 28% higher odds (aOR=1.28; 95% CI, 1.14 to 1.43) and Tucson had 99% higher odds of leaving contact information compared with non-enhanced recruiting regions (aOR=1.99; 95% CI, 1.78 to 2.22) (table 4).

**Table 4:** Adjusted ORs (aORs) generated from a multivariable logistic regression for participant willingness to be contacted for future research by enhanced engagement region, age, sex, race and ethnicity from 17 June 2022 to 28 December 2023

	aOR	95% CI	
		Lower	Upper
Region			
Atlanta	1.255	1.123	1.402
Baltimore	1.171	1.065	1.287
Miami	1.277	1.142	1.427
Tucson	1.986	1.776	2.220
Non-enhanced recruiting region (reference)			
Age			
50–59	1.365	1.295	1.438
60–69	1.379	1.31	1.453
70–79	1.45	1.363	1.544
80+	1.431	1.263	1.621
18–49 (reference)			
Sex			
Male	0.914	0.864	0.968
Female (reference)			
Race			
American Indian/Alaska Native	0.832	0.702	0.986
Asian	0.746	0.655	0.849
Black	1.225	1.100	1.364
Native Hawaiian/ Pacific Islander	0.915	0.66	1.267
Multiracial/mixed race	1.184	1.074	1.306
Prefer not to answer†	0.676	0.611	0.748
White (reference)			
Ethnicity			
Hispanic/Latino	1.198	1.130	1.269
Prefer not to answer	0.593	0.510	0.689
Non-Hispanic/Latino (reference)			

In the multivariable model, compared with white participants, black participants had 23% higher odds of leaving contact information (aOR=1.23; 95% CI, 1.10 to 1.36), and compared with non-Hispanic participants, Hispanic participants had 20% higher odds (aOR=1.20; 95% CI, 1.13 to 1.27) (table 4). Men and those preferring not to leave race or ethnicity information had statistically lower odds of leaving contact information (table 4). Those who preferred not to answer the race question had statistically lower odds of leaving contact information (aOR=0.68; 95% CI, 0.61 to 0.75) as did those who preferred not to answer the ethnicity question (aOR=0.59; 95% CI, 0.51 to 0.69).

## Discussion

Following nationwide and regional efforts to engage black and Hispanic adults in a large online cognitive ageing study, we report increased participation in ELE regions—geographic areas within 60 miles of academic centres in Atlanta, Baltimore, Miami, and Tucson—compared with the rest of the nation. White, non-Hispanic and female participants were over-represented nationally and in ELE regions in terms of MindCrowd participation. Once engaged in MindCrowd, however, black and Hispanic participants were more willing to leave contact information for future research than White and non-Hispanic participants in models adjusting for age, sex and region. Findings underscore ongoing challenges in the representation of racially and ethnically diverse populations in ageing research but suggest that targeted campaigns, employing a combination of online and inperson approaches, can improve willingness to engage these populations in future research.

It is notable that the majority of MindCrowd participants in combined ELE regions were white (78.9%) and non-Hispanic (75.9%), despite these regions being selected for their high underlying proportions of black and Hispanic residents. While there were greater proportions of black and Hispanic participants in ELE compared with non-ELE regions, proportional representation was not achieved.^37^ These findings indicate that the historic challenges of engaging black and Hispanic populations in health research remain and support assertions by other researchers that race and ethnic representation in health research requires extended timeframes for recruiting, higher resourcing costs and strong community partnerships.^13 38^

Consistent with previous studies,^2228^,^30 39^ women participated in Mindcrowd at far higher rates than men nationwide (87.2% female) and in the four ELE regions, which had over 75% female participants in each region (table 1). In ELE regions, researchers included specific activities to recruit men, such as promotion of MindCrowd to male partners and relatives at inperson events, which may account for slight increases in male participation in these regions compared with non-ELE regions. After participating in MindCrowd, however, female and male participants showed only small differences in willingness to leave contact information (42.4% vs 39.5%, table 3), which equated to 9% lower odds of men leaving contact information than women (table 4). These findings suggest that once they participate in MindCrowd, men are only slightly less willing to be contacted for future research than women and that future efforts should focus on increasing initial engagement of men in MindCrowd.

Findings demonstrate that engagement of under-represented communities is possible with intention and resources and that both national and regional campaigns can increase the willingness of black and Hispanic communities to be contacted for future research. Multivariate models accounted for the independent effects of race, ethnicity and ELE region simultaneously and showed significantly higher willingness of black and Hispanic populations to leave contact information even after controlling for the effect of ELE region. This suggests that the national campaigns, with targeted media directed at black and Hispanic communities, also may have had an impact. Findings underscore the previously identified importance of implementing a multipronged approach when recruiting older adults.^40^ A 2021 review on challenges to participation in Alzheimer’s and dementia research among Hispanic individuals cited a lack of culturally appropriate resources and staffing.^41^ Potential solutions include targeted technology-based recruitment strategies for socially isolated communities, investment in building trust, providing incentives for participation, using culturally appropriate recruitment strategies and materials and flexibility in strategies to address barriers to research participation.^42^,^48^

Teams in ELE regions did adapt to the needs of older adults in the community and to potential concerns about public events in confined spaces due to immunocompromised status and concerns about COVID-19. At certain events in Tucson, quiet spaces for taking MindCrowd were designated, facilitating additional participation by adults who may not have otherwise participated. These assistance efforts address previously identified difficulties in engaging older adults in research involving electronic devices, due to lack of familiarity with the medium as well as functional difficulties.^49^ The Miami site held ample virtual events, in addition to inperson events, which allowed participation from a broader base of potential participants, which has also been noted as important in engaging older adults in research, particularly in the context of the recent pandemic.^41 50^

This study was subject to multiple limitations. First, our definition of participation requires access to an electronic device, and while more than 90% Americans over 65 have access to an electronic device across race and ethnicity categories,^51^ access by itself does not connote frequency or sophistication of use, particularly for those in the oldest age categories. Further, access to the MindCrowd website on an electronic device requires proactive engagement on the part of the participant, which might dissuade participants. In ELE regions, this access was enhanced by provision of laptops and iPads for groups of older adults during certain recruitment activities or events. Second, the outcome of willingness to be contacted for future research assumes that individuals participating have an email address. However, given that the vast majority of Americans have an email address, this limitation is likely to be minor. Third, while participating in MindCrowd was possible in Spanish before the site was redesigned in 2022, a Spanish-language version of the redesigned site was reintroduced in August 2023 towards the end of the study period for this analysis. This resulted in fewer Spanish-only speakers (3.4% during the study period) being able to participate, which would bias our study sample to fewer Hispanic participants for whom language was a barrier. Finally, this study did not specify which particular activities in each of the ELE regions led to the greatest gains in participation. This is a topic for future research and evaluation.

In conclusion, this study found higher proportional participation of black and Hispanic individuals in MindCrowd, compared with White and non-Hispanic individuals, in regions with ELE. Further, findings demonstrated increased willingness of all participants to leave contact information, regardless of race or ethnicity, in ELE regions. These findings suggest the importance of a local approach. Further, the study found that, even when controlling for ELE region, black and Hispanic participants were more willing to leave contact information, suggesting that culturally tailored and demographically targeted national media campaigns can also be effective. Future assessment of which specific strategies confer the greatest gains in participation and willingness to be contacted will be important in building an evidence base of effective contemporary strategies. Continued efforts towards representation of black and Hispanic communities in cognitive ageing research are essential to understand specific risk factors for cognitive decline, as well as predictors of resilience and responsiveness to interventions.^52^,^55^

## References

[1] Martinez-Miller EE, Robinson WR, Avery CL (2020). Longitudinal Associations of US Acculturation With Cognitive Performance, Cognitive Impairment, and Dementia. Am J Epidemiol.

[2] O’Bryant SE, Humphreys JD, Smith GE (2008). Detecting dementia with the mini-mental state examination in highly educated individuals. Arch Neurol.

[3] Quiroz YT, Solis M, Aranda MP (2022). Addressing the disparities in dementia risk, early detection and care in Latino populations: Highlights from the second Latinos & Alzheimer’s Symposium. Alzheimers Dement.

[4] Meyer OL, Besser L, Mitsova D (2021). Neighborhood racial/ethnic segregation and cognitive decline in older adults. Soc Sci Med.

[5] Zuelsdorff M, Okonkwo OC, Norton D (2020). Stressful Life Events and Racial Disparities in Cognition Among Middle-Aged and Older Adults. J Alzheimers Dis.

[6] Colby SL, Ortman JM (2014). Current population reports.

[7] Garcia MA, Downer B, Chiu C-T (2019). Racial/Ethnic and Nativity Differences in Cognitive Life Expectancies Among Older Adults in the United States. Gerontologist.

[8] Rajan KB, Weuve J, Barnes LL (2021). Population estimate of people with clinical Alzheimer’s disease and mild cognitive impairment in the United States (2020-2060). Alzheimers Dement.

[9] Chen S, Cao Z, Nandi A (2024). The global macroeconomic burden of Alzheimer’s disease and other dementias: estimates and projections for 152 countries or territories. Lancet Glob Health.

[10] Nandi A, Counts N, Bröker J (2024). Cost of care for Alzheimer’s disease and related dementias in the United States: 2016 to 2060. *NPJ Aging*.

[11] Indorewalla KK, O’Connor MK, Budson AE (2021). Modifiable Barriers for Recruitment and Retention of Older Adults Participants from Underrepresented Minorities in Alzheimer’s Disease Research. J Alzheimers Dis.

[12] Areán PA, Gallagher-Thompson D (1996). Issues and recommendations for the recruitment and retention of older ethnic minority adults into clinical research. J Consult Clin Psychol.

[13] Gallagher-Thompson D, Singer LS, Depp C (2004). Effective recruitment strategies for Latino and Caucasian dementia family caregivers in intervention research. Am J Geriatr Psychiatry.

[14] Godden S, Ambler G, Pollock AM (2010). Recruitment of minority ethnic groups into clinical cancer research trials to assess adherence to the principles of the Department of Health Research Governance Framework: national sources of data and general issues arising from a study in one hospital trust in England. J Med Ethics.

[15] McDougall GJ Jr, Simpson G, Friend ML (2015). Strategies for research recruitment and retention of older adults of racial and ethnic minorities. J Gerontol Nurs.

[16] Forsat ND, Palmowski A, Palmowski Y (2020). Recruitment and Retention of Older People in Clinical Research: A Systematic Literature Review. J Am Geriatr Soc.

[17] Remillard ML, Mazor KM, Cutrona SL (2014). Systematic review of the use of online questionnaires of older adults. J Am Geriatr Soc.

[18] Ashford MT, Camacho MR, Jin C (2023). Digital culturally tailored marketing for enrolling Latino participants in a web-based registry: Baseline metrics from the Brain Health Registry. Alzheimers Dement.

[19] Nash EL, Gilroy D, Srikusalanukul W (2017). Facebook advertising for participant recruitment into a blood pressure clinical trial. J Hypertens.

[20] Wasilewski MB, Stinson JN, Webster F (2019). Using Twitter to recruit participants for health research: An example from a caregiving study. Health Informatics J.

[21] Whitaker C, Stevelink S, Fear N (2017). The Use of Facebook in Recruiting Participants for Health Research Purposes: A Systematic Review. J Med Internet Res.

[22] Sokołowski DR, Pani J, Hansen TI (2024). Participation and engagement in online cognitive testing. Sci Rep.

[23] Ali SH, Foreman J, Capasso A (2020). Social media as a recruitment platform for a nationwide online survey of COVID-19 knowledge, beliefs, and practices in the United States: methodology and feasibility analysis. BMC Med Res Methodol.

[24] Nuño T, Ellingson KD, Chen Z (2024). Increasing Hispanic Participation in Cognitive Research: An Examination of a Decade of Web-Based Recruitment into MindCrowd. Hisp Health Care Int.

[25] Williams KP, Anderson AM (2021). Two Community-Based Strategies to Recruit Black Women in Research. J Urban Health.

[26] Meekes WMA, Ford C, Stanmore EK (2021). Recruitment and retention of older adults in Assisted Living Facilities to a clinical trial using technology for falls prevention: A qualitative case study of barriers and facilitators. Health Soc Care Community.

[27] Staniszewska S, Brett J, Simera I (2017). GRIPP2 reporting checklists: tools to improve reporting of patient and public involvement in research. BMJ.

[28] Lane TS, Armin J, Gordon JS (2015). Online Recruitment Methods for Web-Based and Mobile Health Studies: A Review of the Literature. J Med Internet Res.

[29] Jacomb PA, Jorm AF, Korten AE (2002). Predictors of refusal to participate: a longitudinal health survey of the elderly in Australia. BMC Public Health.

[30] Berron D, Ziegler G, Vieweg P (2022). Feasibility of Digital Memory Assessments in an Unsupervised and Remote Study Setting. Front Digit Health.

[31] Talboom JS, De Both MD, Naymik MA (2021). Two separate, large cohorts reveal potential modifiers of age-associated variation in visual reaction time performance. NPJ Aging Mech Dis.

[32] Hooyman A, Huentelman MJ, De Both M (2023). Establishing the Validity and Reliability of an Online Motor Learning Game: Applications for Alzheimer’s Disease Research Within MindCrowd. Games Health J.

[33] Manson S (2023). IPUMS National historical geographic information system.

[34] US Census Bureau (2022). Quick facts.

[35] Merrill RM (2016). Statistical methods in epidemiologic research.

[36] Rosner B (2011). Fundamentals of biostatistics.

[37] (2023). US census data. https://www.census.gov/quickfacts/fact/table/tucsoncityarizona/PST045223.

[38] Bonevski B, Randell M, Paul C (2014). Reaching the hard-to-reach: a systematic review of strategies for improving health and medical research with socially disadvantaged groups. BMC Med Res Methodol.

[39] Salthouse TA (2019). Attrition in Longitudinal Data is Primarily Selective with Respect to Level Rather than Rate of Change. J Int Neuropsychol Soc.

[40] Degroot L (2022). Lead With the Why: Research Recruitment of Older Adults With HF During COVID-19. J Card Fail.

[41] Massett HA, Mitchell AK, Alley L (2021). Facilitators, Challenges, and Messaging Strategies for Hispanic/Latino Populations Participating in Alzheimer’s Disease and Related Dementias Clinical Research: A Literature Review. J Alzheimers Dis.

[42] Gao L, Green E, Barnes LE (2015). Changing non-participation in epidemiological studies of older people: evidence from the Cognitive Function and Ageing Study I and II. Age Ageing.

[43] Lacey RJ, Wilkie R, Wynne-Jones G (2017). Evidence for strategies that improve recruitment and retention of adults aged 65 years and over in randomised trials and observational studies: a systematic review. Age Ageing.

[44] Nápoles AM, Chadiha LA, Resource Centers for Minority Aging Research (2011). Advancing the science of recruitment and retention of ethnically diverse populations. Gerontologist.

[45] Salazar CR, Hoang D, Gillen DL (2020). Racial and ethnic differences in older adults’ willingness to be contacted about Alzheimer’s disease research participation. Alzheimers Dement (N Y).

[46] Marshall LW, Carrillo CA, Reyes CE (2020). Evaluation of Recruitment of Older Adults of Color into a Community-Based Chronic Disease Self-Management Wellness Pathway Program in Los Angeles County. Ethn Dis.

[47] Raman R, Quiroz YT, Langford O (2021). Disparities by Race and Ethnicity Among Adults Recruited for a Preclinical Alzheimer Disease Trial. JAMA Netw Open.

[48] Aragones A, Hayes SL, Chen MH (2014). Characterization of the Hispanic or latino population in health research: a systematic review. J Immigr Minor Health.

[49] Nkimbeng M, Roberts L, Thorpe RJ (2020). Recruiting Older Adults With Functional Difficulties Into a Community-Based Research Study: Approaches and Costs. J Appl Gerontol.

[50] Moseson H, Kumar S, Juusola JL (2020). Comparison of study samples recruited with virtual versus traditional recruitment methods. Contemp Clin Trials Commun.

[51] Pew Research Center (2024). Internet, broadband factsheet: who uses the internet?. https://www.pewresearch.org/internet/fact-sheet/internet-broadband/#who-uses-the-internet?tabItem=d5edf003-5858-4269-89c5-f2889ecf7951Accessed.

[52] Oh SS, Galanter J, Thakur N (2015). Diversity in Clinical and Biomedical Research: A Promise Yet to Be Fulfilled. PLoS Med.

[53] Stronks K, Wieringa NF, Hardon A (2013). Confronting diversity in the production of clinical evidence goes beyond merely including under-represented groups in clinical trials. Trials.

[54] Rivas-Drake D, Camacho TC, Guillaume C (2016). Just Good Developmental Science: Trust, Identity, and Responsibility in Ethnic Minority Recruitment and Retention. Adv Child Dev Behav.

[55] Rote S, Guitierrez A (2024). Older Mexicans and Latinos in the United States.

